# Lymphoepithelioma-like carcinoma of the bladder: a case report

**DOI:** 10.1186/1752-1947-8-424

**Published:** 2014-12-14

**Authors:** Imad Ziouziou, Tariq Karmouni, Khalid El khader, Abdellatif Koutani, Ahmed Iben Attya Andaloussi

**Affiliations:** Service d’Urologie B, Ibn Sina University Hospital, Faculty of Medicine, Mohamed V Souissi University, Rabat, Morocco

**Keywords:** Bladder, Lymphoepithelioma-like carcinoma

## Abstract

**Introduction:**

Lymphoepitheliomas are malignant epithelial tumors of the nasopharynx characterized by an important lymphoid proliferation at histological examination. Lymphoepithelioma-like carcinoma is a rare tumor of the bladder for which the therapeutic strategy is not clearly defined.

**Case presentation:**

We report the case of a 64-year-old Moroccan man who presented with macroscopic hematuria. Investigations revealed a muscle-invasive lymphoepithelioma-like carcinoma of the bladder. Therefore he underwent a radical cystoprostatectomy with a good outcome.

**Conclusion:**

This case illustrates pathogenic, clinical and therapeutic features of this unusual tumor.

## Introduction

Lymphoepitheliomas are undifferentiated malignant epithelial tumors of the nasopharynx which are recognized histologically by lymphocytic infiltration suggesting an important malignant lymphoma. Tumors of similar histological type have been described in other sites outside of the nasopharynx (lung, stomach, cervix, skin) and are known as lymphoepithelioma-like carcinomas (LELC). The primary urothelial LELC was described for the first time by Zukerberg *et al.* in 1991 [1]. The LELC is a tumor that rarely reaches the urinary tract: there were only 80 cases reported in the bladder, 10 cases in the ureter, and 7 cases in the renal pelvis [2].

We report the case of a patient who had a muscle-invasive LELC of the bladder, and review the literature on this rare condition in order to clarify the clinical and therapeutic features.

## Case presentation

**Figure 1:**
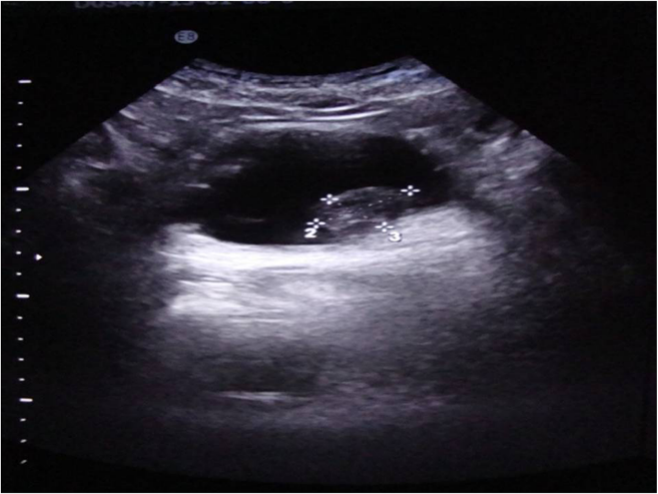
**Echogenic ultrasound image at the left wall of the bladder.**

A 64-year-old Moroccan man had a history of chronic smoking and arterial hypertension treated by losartan and hydrochlorothiazide. He reported a hematuria 2 months ago with blood clots in his urine. His physical examination was normal. Ultrasounds revealed an intravesical echogenic image localized at the left-side wall of his bladder measuring 22mm×26mm (Figure 1).

**Figure 2:**
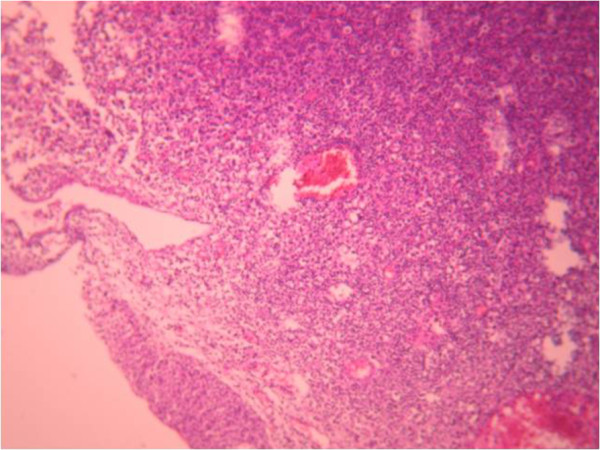
**Massive tumoral infiltration of the stroma with regular superficial urothelial mucosa: hematein and eosin** **×200.**

**Figure 3:**
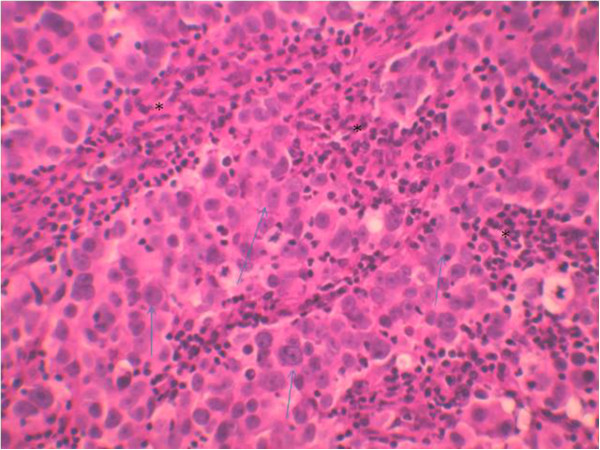
Syncytial clusters of undifferentiated highly vesicular nucleolus nucleus (arrows) cells bathed in abundant lymphoid stroma (*): hematein and eosin **×200.**

**Figure 4:**
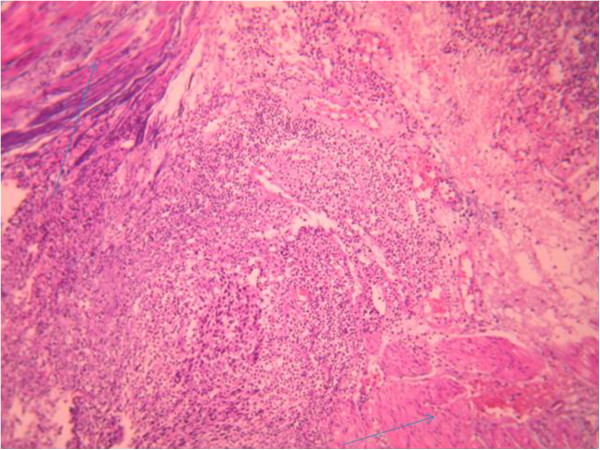
**Massive infiltration of muscle (arrows): hematein and eosin** **×200.**

**Figure 5:**
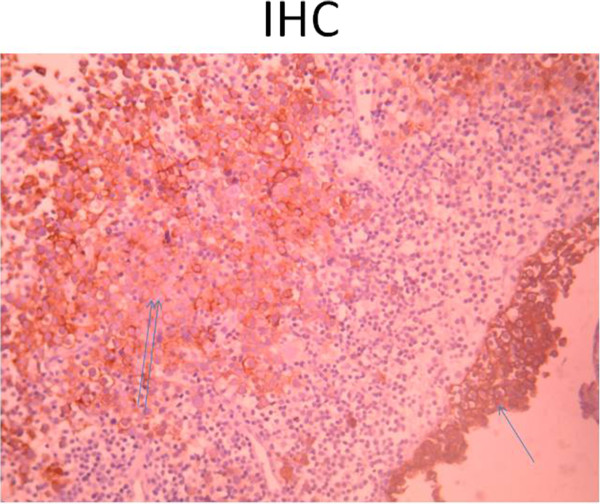
**Anti-cytokeratin antibody (AE1/AE3): positivity on the coating surface (an arrow), less positivity on the tumor cells (two arrows).**

Cystoscopic exploration revealed a solid lesion in the left wall with a large base. A transurethral resection of the bladder tumor was performed. A pathological examination revealed a proliferation of cells with large vesicular nuclei, nucleoli and high mitotic activity. These cells were either arranged in syncytial clusters or isolated within a predominant lymphoid stroma. This proliferation infiltrated (Figures 2, 3 and 4) muscle. An immunohistochemical examination (cytokeratin positivity) confirmed the diagnosis of LELC of the bladder (Figure 5).

Chest and abdominopelvic computed tomography (CT) did not show pelvic lymphadenopathy or secondary location. A radical surgery was then decided without neoadjuvant therapy.

Cystoprostatectomy with Bricker diversion and lymph node dissection were performed. The postoperative course was uneventful.

The pathological examination of the surgical specimen revealed no residual tumor lesion and non-invaded lymph nodes (T0 N0).

After 12 months, the patient was in good condition with normal renal function, and normal chest-abdominopelvic CTs at 6 and 12 months.

## Discussion

LELC of the bladder were classified as a distinct variant of urothelial carcinomas in the classification of the World Health Organization of urothelial tumors in 2004 [3].

The lymphoepithelial carcinoma of the nasopharynx is strongly associated with infection with the Epstein–Barr virus (EBV). However, no risk factor is known for LELC of the bladder [4]; the average age of patients was 65 years with a sex ratio (M:F) of 2:5 [4].

Gulley *et al.* looked for the presence of EBV in LELC of the bladder by *in situ* hybridization techniques: none of 11 cases had viral DNA [5].

Two cases of post-Bacillus Calmette–Guérin (BCG) therapy LELC have been reported by Gastaud *et al.* and Izquierdo-Garcia *et al.*, suggesting a role of immune system activation after the BCG therapy [6, 7].

The clinical presentation of LELC of the bladder does not differ from that of urothelial carcinomas. It is dominated by macroscopic hematuria and irritative voiding disorders.

At endoscopy, the tumor is often unifocal, small, and has a polypoid form [1, 2]. Our case had the same clinical and endoscopic characteristics described in the literature.

LELC is characterized in its pure form by an undifferentiated epithelial tumor with a significant lymphocytic infiltration [8].

Amin *et al.* subdivided LELC – depending on the importance of urothelial carcinoma within the tumor – into [8]: pure LELC, predominant LELC (lymphoepithelial component greater than 50%), and focal LELC (lymphoepithelial component less than 50%).

The predominance of lymphoepithelial component is a good prognostic factor. For some authors, conservative treatment can be achieved even in invasive forms because of their chemosensitivity. Conservative treatment was either endoscopic resection or partial cystectomy followed by adjuvant chemotherapy with cisplatin. Progression-free survival was 47 months as reported by Amin *et al*. [8]. But in this series including 11 patients, only one patient had a predominant LELC form that had chemotherapy with transurethral resection [8]. The number of cases reported and managed by conservative treatment is very low. Most authors recommend radical cystectomy in the case of muscle infiltration [9].

The pathological form of our case was mixed type, in which two components coexisted: lymphoepithelial which was predominant and urothelial.

We opt for radical treatment in patients with urothelial tumor infiltrating the muscle T2 with lymphoepithelial component taking into account the low level of evidence advocating conservative treatment.

Our patient did not have chemotherapy neither neoadjuvant nor adjuvant.

Serrano *et al.* [4] reported a recurrence-free survival in 87.5% of patients who had a pure form and stages T2 or T3. The prognosis of pure forms is linked to the importance of the inflammatory infiltrate and cytotoxic T lymphocytes for two reasons: early onset of symptoms causing patients to seek care, and strengthening the action of substances used in chemotherapy [4].

Most authors advocate the quasi-systematic use of cisplatin-based chemotherapy as adjuvant treatment after transurethral resection or cystectomy [10]. There are even some that offer BCG therapy [11].

## Conclusions

LELC is a rare tumor of the bladder with no therapeutic consensus. Radical treatment seems most appropriate for muscle invasive urothelial tumors with LELC component, especially as literature data, advocating conservative treatment regardless of the tumor stage, has a low level of evidence.

## Consent

Written informed consent was obtained from the patient for publication of this case report and any accompanying images. A copy of the written consent is available for review by the Editor-in-Chief of this journal.

## Abbreviations

BCG: Bacillus Calmette–Guérin
CT: Computed tomography
EBV: Epstein–Barr virus
LELC: lymphoepithelioma-like carcinoma.
